# Movable Bodies in the Synovial Sac at the Articulation of the Trapezium with the Metacarpal Bone of the Thumb

**Published:** 1859-10

**Authors:** T. M. Bentley

**Affiliations:** Jefferson, Wisconsin


*Movable Bodies in the Synovial Sac at the Articulation of the Trapezium with the Metacarpal Bone of the Thumb.*

Jefferson, Wisconsin.

AUSTIN FLINT, JUN.,

*Dear Sir:*—I submit the following:

June 5th, 1859. Mr. B——, aged 25 years, of intemperate habits, general health good, called for medical aid. On examination, two tumors were found; one on the palmar face of the left carpus, and the other on the upper edge of the palmar face of the thumb of the same hand. The tumors were elastic, and easily indented; the one on the thumb would disappear upon pressure, and would reappear by pressing upon the tumor on the wrist. I ascertained, by the patient, the following facts: About six years ago, he was engaged in plowing a piece of new, rough ground, and at night thought his wrist was strained, as it was painful and swelled. It remained painful for some months. He used a compress, bandage, etc., without relief. For the last five years it has not been painful, although the flexor tendons of the middle and ring fingers are contracted, so that he cannot straighten them.

I proposed an operation, to which he readily consented. I accordingly made two incisions, half an inch in length and three quarters of an inch in depth; one in the thumb, and one in the palmar face of the wrist, over the flexor tendon of the ring finger; out gushed a large mass of irregular-shaped transparent, tenacious, elastic bodies, smooth, of various shapes, as spherical, oval, round, and flat. These, in shape, might be compared to the pebbles in a brook. They were contained in three distinct cells, and passed through sinuses over the palm of the hand and to the side of the thumb. A probe was introduced, to the distance of 4½ inches, in one of those sinuses. They were collected principally over and around the flexor tendons of the hand at the wrist.

Drs. A. H. Vanostrand and W. W. Reed examined the bodies, at my office soon after the operation. There was very little fluid contained in the tumor, and it seemed to be of a sero-lymph-like character. 6th. Patient suffered severe pain until 3 o'clock this morning. The application of wet cloths procured three hours' sleep. At 8 o'clock this morning I injected, with a large syringe, a weak solution of caustic potassa, which caused severe pain for a few minutes, and then perfect rest for three and a half hours, during which time he slept soundly. He is now walking about with his hand in a sling. Yours truly,

T. M. BENTLEY.

In a note appended to the history of this interesting case, we are desired to express an opinion upon the character of the tumor, and the propriety of the operation. This case is one of exceeding rarity and interest; and the history is perfectly complete, with the exception of a microscopic examination of the bodies. From the description of these bodies, we have no doubt but that they were what are called inter-articular cartilages, or movable bodies in the synovial cavities. The synovial sacs about the wrist are quite numerous; but the disease appears to have been situated in the one at the articulation of the trapezium with the metacarpal bone of the thumb. The origin of these bodies has never been quite satisfactorily explained, though they were described as early as the middle of the sixteenth century by Paré. Some of them are considered by Rokitansky as organized fibrinous clots, and some of them he thinks arise by an inflammatory action from the adjacent cartilages, to which they are first attached by a pellicle, which afterwards disappears, leaving them free in the cavity, and apparently covered by a delicate serous membrane. They occur, according to all observers, most frequently in the knee-joint, where they sometimes are found as large as the patella even; and in this situation it is thought by surgeons to be extremely hazardous to interfere with the knife. They are found, however, in other joints, though rarely or never in the ball and socket articulations. Rokitansky alludes to their occurrence in the peritoneal and other serous cavities. There may be one or two of great size, or a large number of small ones; though Prof. Gross, in his recent work on surgery, speaks of eighteen as the largest number which he has ever known to exist in a single sac. We should be obliged to our correspondent if he would give us the exact number which he found in his case. It is unnecessary to enter into a description of these bodies, as that given in the notes of the case corresponds almost exactly with the description of inter-articular bodies in the books.

As regards the propriety of surgical interference, we consider the operation performed as by all means justifiable, being, of course, the only means of giving permanent relief. In addition to the rarity of these formations in any situation, the large number and the situation make the case particularly interesting and unique.

